# Integrating a Community-Based Health Information System with a Patient-Centered Medical Home to improve care of patients with hypertension: a longitudinal observational study protocol

**DOI:** 10.1186/s12913-024-11012-0

**Published:** 2024-04-27

**Authors:** Unab I. Khan, Sabeen Shah, Shankar Viswanathan, Asra Qureshi, Yasmeen Noornabi, Mahnoor Niaz, Judith Wylie-Rosett

**Affiliations:** 1 Department of Family Medicine, Aga Khan University, Karachi, Sindh Pakistan; 2 Department of Epidemiology & Population Health, Albert Einstein College of Medicine, Bronx, New York USA

**Keywords:** Hypertension, Disease management, Primary health care, Community-based health information system, Patient-centered medical home

## Abstract

**Background:**

The primary healthcare system in Pakistan focuses on providing episodic, disease-based care. Health care for low-middle income communities is largely through a fee-for-service model that ignores preventive and health-promotive services. The growing burden of cardiovascular illnesses requires restructuring of the primary health care system allowing a community-to-clinic model of care to improve patient- and community-level health indicators.

**Methods:**

We propose a model that integrates a Patient-Centered Medical Home (PCMH) with a Community-Based Health Information System (CBHIS) using hypertension (HTN) as an example. This protocol describes the integration and evaluation of the PCMH-CBHIS infrastructure through a population-based, observational, longitudinal study in a low-middle income, urban community in Pakistan. Participants are being enrolled in CBHIS and will be followed longitudinally over two years for HTN outcomes. A mixed-methods approach is adopted to evaluate the process of integrating PCMH with CBHIS. This involves building partnerships with the community through formal and informal meetings, focus group discussions, and a household health assessment survey (HAS). Community members identified with HTN are linked to PCMH for disease management. A customized electronic medical record system links community-level data with patient-level data to track changes in disease burden. The RE-AIM evaluation framework will be used to monitor community and individual-level metrics to guide implementation assessment, the potential for generalization, and the effectiveness of the PCMH in improving HTN-related health outcomes. Ethical clearance has been obtained from the Ethics Review Committee at Aga Khan University (2022-6723-20985).

**Discussion:**

This study will evaluate the value of restructuring the primary care health system by ensuring systematic community engagement and measurement of health indicators at the patient- and community-level. While HTN is being used as a prototype to generate evidence for the effectiveness of this model, findings from this initiative will be leveraged towards strengthening the management of other acute and chronic conditions in primary care settings. If effective, the model can be used in Pakistan and other LMICs and resource-limited settings.

## Background

Pakistan, with a population of nearly 240 million, faces a double burden of communicable and non-communicable diseases (NCDs) [1]. Deaths due to NCDs have increased from 46% in 2000 to 60% in 2019 [2], and cardiovascular disease (CVD) (including ischemic heart disease and stroke) remains the leading cause of these deaths [3]. Hypertension (HTN) is the leading modifiable CVD risk factor, and one in four adults is diagnosed with HTN [4]. In addition, blood pressure (BP) control rates as low as 6.4% have been reported at the primary care level [5], contributing to more than half of CVD-related morbidity [6].

The fragmented healthcare system in Pakistan relies heavily on the private sector, especially in urban areas, where 70% of care is delivered at private clinics and hospitals with a fee-for-service model [7]. With rising inflation, health care is becoming increasingly expensive, leading to episodic, disease-specific care with little or no focus on health promotion and disease prevention. The financial burden of chronic disease management creates additional challenges to compliance, further contributing to morbidity and mortality. This is especially true for low-middle income families, who are largely ineligible for government-funded health programs and rely on the unmonitored and unregulated private-sector for healthcare [8].

Low-middle income families in Pakistan are defined as households with the ability to spend $2-4/person/day [9, 10]. These families have a secure income source, can spend beyond essential goods, and can pay for health and education. However, if struck by catastrophic health crises, the financial implications of illness can plunge them below the poverty line [11]. In 2011, low-middle income families constituted 55% of households, with an average growth of 9.4% per year in the last decade [9]; with an estimated 84 million people [12].

The Aga Khan University’s (AKU) health research mandate is to improve healthcare through contextual solutions. In this vein, the University has funded a primary health center, Family Medicine Health Center (FMHC) to create a proof-of-concept model that, if effective, can be replicated by other health systems. Using the principles of a Patient-Centered Medical Home (PCMH), FMHC provides accessible, cost-effective, quality care in the community setting [13–15]. In addition, it integrates with a Community-Based Health Information System (CBHIS), to examine the effectiveness of the model on health outcomes at the patient and community level.

In the absence of a national integrated health information system that collects comprehensive morbidity and mortality parameters, we are unable to make data-driven decisions at the population level [16]. Thus establishing a CBHIS for continuous monitoring will be essential to test the effectiveness of the new model [17, 18]. A CBHIS has been created for the catchment population around FMHC to measure the disease burden and obtain baseline estimates that will enable us to assess change at the individual and community levels.

**Fig. 1:**
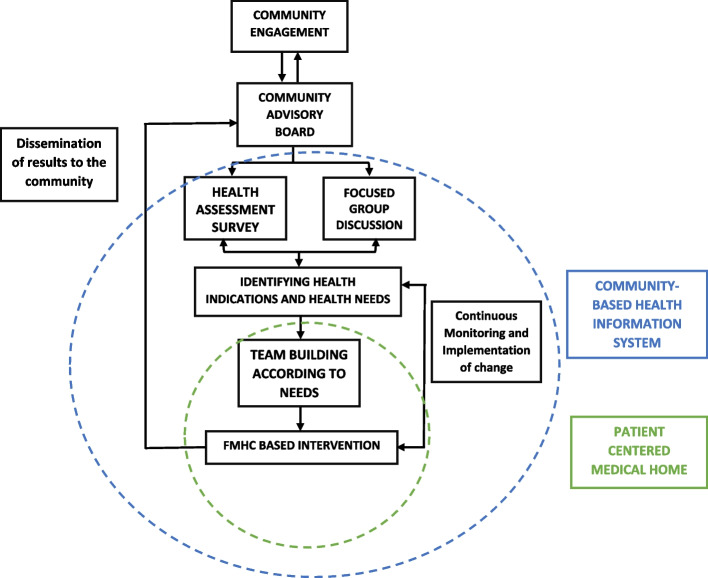
Integrating PCMH to Community-Based Health Information System (CBHIS) for delivering primary health care at FMHC

Figure 1, denotes the juxtaposition of the CBHIS with a PCMH model at FMHC, illustrating the steps undertaken to create a system of ongoing bilateral communication between the community and the FMHC team. A series of dialogues between the community and the FMHC team have led to formation of a Community Advisory Board (CAB), that supports planning and conducting focus group discussions (FGDs) with community members and initiate a family-level health assessment survey (HAS) for all families within a 0.5-kilometer radius of FMHC. Information from the initial HAS and FGDs will be the basis of the CBHIS, and findings from annual surveys will be leveraged to assess the impact of FMHC-based interventions at the community level. In addition, subsequent FGDs will allow us to understand the context of the responses in HAS. An electronic medical record (EMR) system is being used to connect the patient-level data from FMHC to community-level data in CBHIS.

## Methods/ design

### Research aim

We propose to integrate and evaluate the PCMH-CBHIS infrastructure to improve the health outcomes of patients diagnosed with HTN within the catchment area of FMHC.

### Study design

This is a population-based, observational, longitudinal study. Participant enrolment in CBHIS is through convenience sampling of the catchment population. We will use a mixed-methods approach to evaluate the process of integrating and evaluating the PCMH – CBHIS infrastructure.

### Study setting

FMHC is located in District Central of Karachi. The district’s area is 69 km^2^ with a population density of 43,063.51 persons per km^2^ [19]. Most people live in apartment complexes. Our catchment population includes families residing within a 0.5-kilometer (km) radius of FMHC with an approximate population of 34,679 individuals [20].

### Study period

The preliminary implementation and evaluation will be conducted over 30 months between June 2022 to November 2024.

### Sample estimates

Based on 2017 census, the population of District Central is 2,971,382 [19]. As 45% of Pakistan’s population is children (under 18 years of age) [21], we estimate that 1,634,260 adults (>18 years) are residing in District Central with a population density of 23,685 adults/km2. The catchment area is estimated to have 19,074 adults(>18 years) [20]. Considering the prevalence of HTN in urban Pakistan as 18% [21], we assume that an estimated 3,433 adults in the catchment area will have HTN. We expect to serve 20% of patients with HTN at FMHC, thus aiming to improve health outcomes for an estimated n:700 adults with HTN.

#### Creation of a Community-Based Health Information System

A multidisciplinary team-based approach is being adopted to create and maintain the CBHIS, with community involvement vital to optimizing and implementing ongoing activities.

(A) Community Engagement

*Formal and informal meetings:* Creation of a CBHIS and its utility in health provision at FMHC has been introduced to community leaders, faith leaders, business leaders, and other key stakeholders in the catchment area. These meetings have allowed us to understand the community's cultural dynamics, current options of care, their openness to try alternative options, and identify ways of ensuring maximal community participation. The formation of a CAB has helped with ongoing bidirectional communication, ensuring participation by families in creating and maintaining the CBHIS and informing them about services at FMHC. As stakeholders, CAB members also provide inputs into study methods, such as the feasibility of hiring community members for conducting the HAS and identifying volunteer families for piloting HAS.

*Focus group discussions:* To better understand the community's health-related needs, exploratory FGDs have been conducted to understand: 1) the community's perception of quality health care, 2) self-perception of health needs, 3) self-perception of health status, 4) barriers to access care, 5) attributes of a facility perceived to offer quality care, 6) self-perception about role of continuity-of-care and comprehensive care in quality health care, and 7) the willingness and paying power for quality care . Working with community leaders a private space accommodating 15-20 people was arranged. Respecting cultural nuances, separate FGDs for men, women, youth, and key stakeholders were conducted. Subsequent FGDs will be conducted amongst a representative sample for insights on FMHC-based interventions.

(B) Health Assessment Survey (HAS)

The HAS is the basis of the CBHIS, which serves as a platform to understand the disease burden of the community and guide in prioritizing service delivery and prevention efforts at FMHC. Survey questions have been derived from existing national standardized surveys and modified to cultural context [22–24]. A door-to-door survey of all consenting families in the catchment area is being conducted at baseline. A household has been identified as "Person or group of related and unrelated persons who live together in the same dwelling unit(s) or in connected premises, who acknowledge one adult member as head of the household, and who have common arrangements for cooking and eating" [23]. However, if there is subletting, or the extended family has separate income and expenses, each is considered an independent family within the apartment**.** The survey includes family level (demographics, family income, healthcare expenditure) and individual-level data (such as medical history, healthcare utilization, anthropometrics (height, weight, waist circumference), BP, and perception of health). Subsequent annual surveys will be conducted to evaluate changing demographics and disease trends.

*Survey administration:* The survey is being conducted in Urdu, the local language. The survey was pilot tested in the community to determine face validity. Piloted and ERC-approved versions of HAS were incorporated as an e-questionnaire in REDCap [25]. Trained community health workers (CHWs) record responses on Android tablets. After informed consent, the survey begins with the head of the family, followed by all other consenting members.

*Selection and role of community health workers:* With input from CAB members, CHWs have been recruited from outside the catchment area who , 1) map the catchment area, 2) conduct surveys, 3) strengthen connections between the community and FMHC team through care coordination, such as by helping community members with HTN access FMHC services, 4) provide informal counseling on healthy behaviors, and 5) provide input to improve survey response rate.

*Training of community health workers:* A two-day training was conducted to enhance CHWs' competency in 1) soft skills such as respectful communication with peers and community members, time management, etc. 2) technical skills such as the use of REDCap for HAS administration, 3) clinical skills to obtain BP and anthropometric measurements, and 4) knowledge about evidence-based algorithms modified for community settings to facilitate prompt identification of uncontrolled BP and early linkage to FMHC. The research team monitors data for accuracy, completeness, and consistency every week and provides feedback to the CHWs.

*Data Analysis of HAS:* We will use descriptive statistics to understand 1) the burden of HTN and other NCDs in the community, 2) HTN control rates, 3) the distribution of CVD risk factors (such as smoking, obesity, and physical activity), and 4) health care utilization (outpatient and hospitals settings). Additionally, sub-group analyses will be performed using appropriate statistical tests (e.g., ANOVA, Chi-square) comparing demographics, health expenditure, access to care, and presence of comorbid illnesses among patients with 1) controlled and uncontrolled HTN, and 2) patients at FMHC and FMHC non-users.

(C) Optimization of Services at Family Medicine Health Center

Delivery of patient - centered care is ensured by offering evidence-based clinical care through a shared decision making process [26]. Clinical team delivers onsite educational sessions to improve health literacy related to HTN and other co-morbid illnesses. [26] These interactions incorporate environmental and social determinants of health to stimulate self-management in HTN through a heart-healthy diet, reduction in sodium intake, increased physical activity, reduction in overweight and obesity, as well as improved knowledge of HTN risk factors. The on-site, small-group educational sessions are publicized to the wider community by CHWs and CAB members. Additionally, CHWs invite HAS participants identified with elevated BP or HTN over the phone or in-person during routine field visits [27]. If needed, specialty referrals are arranged at AKU’s community hospital in Karimabad which is 1.5 km from FMHC.

The research team and the clinical team at FMHC meet regularly to share insights about community-level and patient-level issues.

*Training for the clinical team:* A two-day training of FMHC doctors and nurses was conducted to enhance clinical knowledge about HTN management and CVD risk assessment based on World Health Organization (WHO) recommendations [28]. Training for doctors included various aspects of HTN care to administer age – and sex-specific care packages in an opportunistic manner that support: 1) diagnosis of HTN, 2) CVD risk assessment and stratification, 3) treatment initiation using appropriate antihypertensive drug class, 4) counseling about lifestyle change, modified to cultural context, 5) medication dose adjustment, 6) recognition of red flags and points of referral, and 7) introduction to the monitoring and evaluation indicators.

The scope of training for nurses covered 1) standardized BP and weight measurement, 2) diagnosis of HTN, 3) counseling about lifestyle change, modified to cultural context, 4) early recognition of red flags and points of referral, and 5) introduction to the monitoring and evaluation indicators. Ongoing capacity-building sessions will be geared towards refresher training and the inclusion of new knowledge and skills to meet evolving needs of FMHC services.

*Health information technology:* FMHC has a customized electronic medical record (EMR) that uses ICD-11 codes for standardized data entry [29]. This maximizes the potential of EMR to provide structured data for analyzing disease trends and recognizing upcoming health issues at individual, family, and community levels. The EMR incorporates the individual patient's HAS data from CBHIS into their medical record using a unique survey number. This allows the healthcare team access to survey data to guide personalized counseling based on identified barriers to care to enhance self-efficacy in HTN management. EMR includes the cost of different brands of antihypertensive medications to allow physicians to identify affordable medications based on patients' paying power [30].

*Analysis of EMR data:* A dashboard provides access to data in real time to facilitate interpretation and ensure the availability of information for auditing and quality improvement. Patient-level data is being analyzed to follow trends of HTN control and how this varies with other health parameters such as body mass index (BMI) and biochemical measurements (such as serum creatinine level, cholesterol level, and fasting glucose level). Additionally, EMR-generated reports summarize the utilization of FMHC services (such as initial versus follow-up ratio, clinic no-shows and patient waiting time, etc.) to improve efficiencies in clinic workflow. Data will be analyzed at six-month intervals to track change in health outcomes of patients diagnosed with HTN.

(D) Evaluation of Integrated Community-Based Health Information System and Patient-Centered Medical Home Model

We will use the RE-AIM framework to evaluate the effectiveness of this integrated model on HTN management at the patient and community levels. RE-AIM's five domains (Reach, Effectiveness, Adoption, Implementation, and Maintenance) have been used in various settings to evaluate the impact of both clinical and community-based interventions, including the management of disease-focused interventions [31–33]. Both quantitative and qualitative data will be collected to help with the monitoring and evaluation of the program. Process and outcome indicators will encompass community-level, patient-level, and FMHC-level data, such as site audits leading to identifying and addressing HTN management gaps.

#### Operational definitions

*Patient with hypertension:* individuals ≥ 18 years with a diagnosis of HTN.

*Uncontrolled hypertension:* based on WHO guidelines; BP readings ≥ 140/90 in individuals with a diagnosis of HTN; ≥ 130/80 for patients with HTN coexisting with CVD or diabetes [28].

*Elevated blood pressure:* single reading of ≥ 140/90 for individuals (≥ 18 years) with no prior diagnosis of HTN.

*Community:* Catchment population within a 0.5-kilometer radius of FMHC.

*CBHIS participants:* Community members enrolled in HAS.

*FMHC patients:* CBHIS participants who establish care at FMHC.

*FMHC non-users:* CBHIS participants who do not establish care at FMHC.

#### RE-AIM domains and key questions

A mixed methods approach will be used to measure outcomes related to HTN management at FMHC. Outcomes will be assessed through annual HAS data, FGDs, EMR reports, and financial data. The following key questions under each dimension of RE-AIM will be assessed periodically as mentioned in Table 1.

- *Reach*
  - What is the extent of the community’s participation in CBHIS?
  - What is the utilization of FMHC services by CBHIS participants?
  - What are the barriers to accessing care at FMHC?
- *Effectiveness*
  - What is the impact of the PCMH-CBHIS model on the management of HTN amongst FMHC patients?
  - What proportion of patients with HTN received comprehensive care at FMHC?
  - What are the barriers to HTN control amongst CBHIS participants registered at FMHC?
- *Adoption*
  - What is the CHWs' capacity for ongoing community engagement activities with CBHIS participants?
  - What is the compliance of the FMHC clinical team to HTN management guidelines?
- Implementation
- What is the fidelity of CHWs to follow project-specific standard operating procedures?
- What is the fidelity of the FMHC clinical team to follow HTN guidelines?
- *Maintenance*
  - What is the financial impact of implementing the PCMH-CBHIS model of care for patients with HTN visiting FMHC?

**Table 1:** Process and Evaluation Indicators using RE-AIM Framework

**RE-AIM dimension**	**Key question**	**Settings**	**Outcome measures**	**Timeline for monitoring and evaluation**								**Data source**
				**Y1**				**Y2**				
				**Q1**	**Q2**	**Q3**	**Q4**	**Q1**	**Q2**	**Q3**	**Q4**	
**Reach**	What is the extent of the community’s participation in CBHIS?	Community	• % of families residing in the catchment area who participated in CBHIS • % of CBHIS participants with a diagnosis of HTN • % of CBHIS participants with uncontrolled HTN • % of CBHIS participants with elevated BP		✓		✓		✓		✓	HAS
	What is the utilization of FMHC services by CBHIS participants?	FMHC	• % of CBHIS participants with HTN registered at FMHC • % of CBHIS participants with uncontrolled HTN registered at FMHC • % of CBHIS participants with elevated BP registered at FMHC				✓				✓	HAS EMR
	What are the major barriers to accessing care at FMHC?	FMHC & Community	• Barriers to accessing care at FMHC by CBHIS participants with HTN				✓				✓	FGDs
**Effectiveness**	What is the impact of the PCMH-CBHIS model of care on the management of HTN at FMHC?	FMHC	• % of newly diagnosed patients with HTN from CBHIS • % of newly diagnosed patients prescribed antihypertensive medications within 90 days of identification of elevated BP by CHWs • % of FHMC patients diagnosed with HTN with ≥ 2 annual documented follow-up visits				✓				✓	EMR
	What are the barriers to HTN control amongst CBHIS participants registered at FMHC?	FMHC & Community	• Barriers to HTN control amongst patients at FMHC				✓				✓	FGDs
	What proportion of patients with HTN received comprehensive care at FMHC?	FMHC	% of patients with HTN with documented evidence of annual: • Diabetes mellitus screening • Depression screening (PHQ-2 score) • Renal function testing • Dyslipidemia screening				✓				✓	EMR
**Adoption**	What is the CHWs' capacity for ongoing community engagement activities with CBHIS participants?	Community	• Number of educational sessions organized by CHWs • % of CBHIS participants who attend educational sessions • Number of data dissemination sessions organized by CHWs with CAB				✓				✓	Attendance log sheets of the session
	What is the compliance of the FMHC clinical team to HTN management guidelines?	FMHC	% of patients receiving guideline-based care: • Counselling on ≥ 1 behavioral risk factor for healthy lifestyle counselling • Choice of antihypertensive medications for newly diagnosed HTN cases				✓				✓	EMR
**Implementation**	What is the fidelity of the CHWs to follow project-specific SOPs?	Community	• % of appropriate referrals generated by CHWs workers for CBHIS participants with uncontrolled HTN • % of appropriate referrals generated by CHWs for CBHIS participants with elevated BP				✓				✓	HAS
	What is the fidelity of the FMHC clinical team to follow HTN management guidelines?	FMHC	• Consistency in prescribing ACE inhibitors/ ARBs to patients with diabetes and diagnosis of HTN • Consistency in annual screening for nephropathy amongst patients with uncontrolled HTN • % of patients with documented evidence of annual CVD risk assessment • Consistency in initiating statins for FMHC patients with WHO CVD risk score ≥20%				✓				✓	EMR
**Maintenance**	What is the financial impact of implementing the PCMH-CBHIS model of care for patients with HTN visiting FMHC?	FMHC	• Annual implementation cost HTN project at FMHC				✓				✓	Administrative data (revenue versus expenditure reports)

*Abbreviations*: *ACE inhibitors* Angiotensin-Converting Enzyme inhibitor, *ARB* Angiotensin-Receptor Blocker, *BP* Blood Pressure, *EMR* Electronic Medical Record, *FMHC* Family Medicine Health Centre, *CAB* Community Advisory Board, *CBHIS* Community Based Health Information System, *CHWs* Community Health Workers, *HTN* Hypertension, *BMI* Body Mass Index, *WC* Waist Circumference, *HAS* Health Assessment Survey

## Discussion

Our PCMH-CBHIS integrated model of care at FMHC uses a community-to-clinic approach to identify and manage, patients with HTN. It demonstrates a well-coordinated effort between community stakeholders, low-resource health care staff (CHWs), and the clinical team to augment the system's performance in improving health indicators at the individual and population level. Existing literature provides support for the PCMH model to enhance clinical outcomes and reduce healthcare expenditures for patients with chronic illnesses [34–36]. Learning from landmark studies such as the COBRA trial [37], our model builds on community engagement through CAB and CHWs since inception, inculcating a sense of ownership and empowerment that ensure sustainability [38, 39]. In addition, ongoing community engagement through surveys and FGDs will allow us to explore further avenues for optimizing care at the individual, family, and community levels.

## Strengths

Major strengths of this study are the use of an implementation science framework to evaluate the effectiveness of the model in a real-world setting. The development of a customized EMR supports aligning clinic- and community-based activities. Additionally, the process integrates a comprehensive health assessment survey to assess the disease burden at the community level to guide the prioritization of health services and prevention efforts at the health facility.

## Limitations

It is possible that some community members may migrate from the catchment area, limiting the longitudinal assessment of hypertension management. In addition, with a patient-driven model, we cannot predict how many community participants would choose the health facility for ongoing care.

In conclusion, the proposed model can proactively guide changes in the health outcomes of patients with HTN. By generating evidence on the effectiveness and addressing health system-related barriers, this model will act as a proof-of-concept for strengthening the management of NCDs in primary care settings across Pakistan and other developing countries with fee-for-service health models. While we will use HTN as a prototype condition to measure health outcomes, the integrated PCMH-CBHIS model could be used to assess the effectiveness of primary-care interventions for other chronic and acute health conditions.

## Data Availability

The datasets generated and/or that will be analysed during the current study will be available from the corresponding author on reasonable request.

## Abbreviations

AKU: Aga Khan University
BMI: Body Mass Index
BP: Blood Pressure
CAB: Community Advisory Board
CBHIS: Community-Based Health Information System
CHW: Community Health Worker
CVD: Cardiovascular Disease
EMR: Electronic Medical Record
ERC: Ethical Review Committee
FGD: Focus Group Discussion
FMHC: Family Medicine Health Center
HAS: Health Assessment Survey
HTN: Hypertension
NCD: Non-communicable Disease
PCMH: Patient Centered Medical Home
SOPs: Standard Operating Procedures
WHO: World Health Organization
